# Canavanine resistance mutation *can1-1* in *Schizosaccharomyces pombe* is a missense mutation in the ubiquitin ligase adaptor gene *any1*

**DOI:** 10.17912/micropub.biology.000538

**Published:** 2022-03-14

**Authors:** Yu-Sheng Yang, Shao-Kai Ning, Xiao-Hui Lyu, Fang Suo, Guo-Song Jia, Wen Li, Li-Lin Du

**Affiliations:** 1 National Institute of Biological Sciences, Beijing, China; 2 Tsinghua Institute of Multidisciplinary Biomedical Research, Tsinghua University, Beijing, China

**Figure 1. The can1-1 mutation is a missense mutation in the any1 gene:** **(A)** Whole-genome sequencing showed that the *can1-1* mutation is not in the *SPBC18H10.16* gene but in the *any1* gene. Mapped sequencing reads are visualized as grey lines. Sequence variants are highlighted by color. WT, wild type. **(B)** Wild-type cells were transformed with an *any1-R175C* PCR product and transformant clones were selected on a canavanine-containing plate. Transformation with an *any1 ^+^* PCR product served as a control, which is expected to only yield spontaneously occurring canavanine-resistant clones. **(C)** Chromosomal locations of *SPBC18H10.16* and *any1* . **(D)** Phylogenetic relationships of *S. cerevisiae* and *S. pombe* proteins containing the “amino acid/polyamine transporter I” (G3DSA:1.20.1740.10) domain. Branch labels are the SH-aLRT support value (%) and the UFBoot support value (%) calculated by IQ-TREE. Scale bar indicates 0.6 substitutions per site. The names of *S. cerevisiae* and *S. pombe* proteins are colored black and red, respectively. **(E)** Structures of Any1-Cat1 complexes predicted by AlphaFold-Multimer. For clarity, only the region of Cat1 involved in Any1 binding (residues 40-52) and the structured region of Any1 (residues 17-338) are shown. The residue at position 175 of Any1 is shown in a surface representation and colored in red. **(F)** Schematic of a model explaining how the *any1-R175C* ( *can1-1* ) mutation causes canavanine resistance.

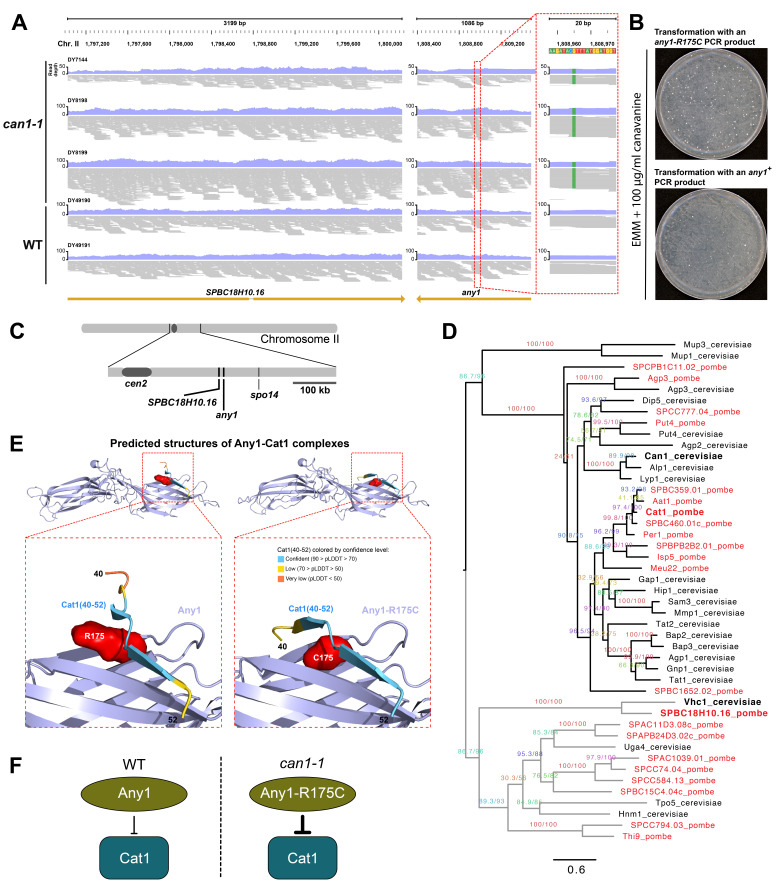



## Description

Canavanine is a toxic arginine analog first isolated from jack bean ( *Canavalia ensiformis* ) (Kitagawa and Yamada 1932). Because the toxicity of canavanine depends on its cellular uptake mediated by plasma membrane arginine transporters, the isolation of canavanine-resistant mutants in model microorganisms yielded pioneering discoveries on arginine transporter genes (Schwartz *et al.* 1959; Grenson *et al.* 1966). In the budding yeast *Saccharomyces cerevisiae* , all canavanine-resistant mutants isolated are loss-of-function mutants of the arginine transporter gene *CAN1* (Whelan *et al.* 1979; Hoffmann 1985). Canavanine-based counter selection of *CAN1* has been widely used as an assay for studying mutagenesis and genome instability in *S. cerevisiae* (Drake 1991; Tishkoff *et al.* 1997; Hackett *et al.* 2001; Lang and Murray 2008; Kim *et al.* 2011; Jiang *et al.* 2021).

In the fission yeast *Schizosaccharomyces pombe* , canavanine-resistant mutants were first isolated at the Institute of General Microbiology, University of Bern, Switzerland in the 1970s and the phenotype-causing mutations in these mutants were mapped to two loci, designated *can1* and *can2* (Kohli *et al.* 1977). A mutation at the *can1* locus, *can1-1* , was shown to cause both canavanine resistance and reduced arginine uptake (Fantes and Creanor 1984). The canavanine resistance phenotype of the *can1-1* mutant can be reversed by the expression of the *S. cerevisiae* *CAN1* gene (Ekwall and Ruusala 1991). These observations have led to the assumption that *can1-1* is a loss-of-function mutation in an *S. pombe* arginine transporter gene (Fantes and Creanor 1984; Ekwall and Ruusala 1991). In PomBase (Lock *et al.* 2019), the gene name *can1* has been assigned to the gene *SPBC18H10.16* . This gene has not been subjected to in-depth experimental characterization. The nature of the *can1-1* mutation has not been reported.

Here, we applied whole-genome sequencing to identify mutations in three *S. pombe* *can1-1* mutant strains. To our surprise, no mutations were found in the gene *SPBC18H10.16* (Figure 1A). Instead, the only mutation found in all three *can1-1* strains but not in two wild-type strains is a G-to-A mutation at position 1808965 of chromosome II in the PomBase reference genome, which results in an arginine-to-cysteine (R175C) missense mutation in the gene *any1* ( *SPBC18H10.20c* ) (Figure 1A). Transformation experiments showed that the *any1-R175C* mutation can indeed cause canavanine resistance (Figure 1B). This unexpected finding led us to investigate the possible reason behind the assignment of the name *can1* to *SPBC18H10.16* . We were not able to find any published evidence directly demonstrating that the *can1* locus corresponds to *SPBC18H10.16* . In the last comprehensive genetic map of *S. pombe* (Munz *et al.* 1989), the *can1* locus was mapped to the interval between the centromere of chromosome II ( *cen2* ) and the *spo14* gene on the right arm of chromosome II. Both *SPBC18H10.16* and *any1* are within this interval and are only 8.2 kb away from each other (Figure 1C). According to PomBase, the *cen2* – *spo14* interval contains 106 protein-coding genes, among which *SPBC18H10.16* is the only one encoding a protein with similarity to amino acid transporters. Thus, we surmise that the PomBase gene name assignment for *SPBC18H10.16* was probably based on the genetic mapping position of the *can1* locus and the assumption that this locus encodes an amino acid transporter.

The similarity between the SPBC18H10.16 protein and experimentally characterized amino acid transporters is limited to an approximately 450-amino-acid domain named “amino acid/polyamine transporter I” (CATH-Gene3D database entry G3DSA:1.20.1740.10) (Sillitoe *et al.* 2021). To clarify whether SPBC18H10.16 is an amino acid transporter, we performed a phylogenetic analysis (Figure 1D). This analysis showed that *S. cerevisiae* and *S. pombe* proteins containing the G3DSA:1.20.1740.10 domain fall into two families. In the family of which *S. cerevisiae* Can1 is a member (black branches in the phylogenetic tree in Figure 1D), all experimentally characterized family members are amino acid transporters. In contrast, in the other family of which SPBC18H10.16 is a member (grey branches in the phylogenetic tree in Figure 1D), all experimentally characterized members are transporters of non-amino-acid compounds (Vhc1, Uga4, Tpo5, Hnm1 of *S. cerevisiae* are transporters of metal and chloride ions, gamma-aminobutyrate, polyamine, and choline, respectively, and Thi9 of *S. pombe* is a transporter of thiamine). In particular, this analysis showed that SPBC18H10.16 is an ortholog of *S. cerevisiae* Vhc1, a cation–chloride cotransporter localized at the vacuole membrane (Petrezselyova *et al.* 2013). Consistent with this orthology, SPBC18H10.16 has been shown to localize at the vacuole membrane in *S. pombe* (Liu *et al.* 2015). Thus, *SPBC18H10.16* encodes not a plasma membrane amino acid transporter but a vacuole membrane cation–chloride cotransporter. We propose that *SPBC18H10.16* should be renamed *vhc1* .

The *any1* gene in *S. pombe* encodes an α-arrestin. α-arrestins are ubiquitin ligase adaptors that target plasma membrane transporters for ubiquitination by the NEDD4/Rsp5 family ubiquitin ligases, and thereby reduce the levels of transporters at the plasma membrane through altering their trafficking (Alvarez 2008; Lin *et al.* 2008; O’Donnell and Schmidt 2019; Kahlhofer *et al.* 2021). In *S. pombe* , Any1 negatively regulates two plasma membrane amino acid transporters Aat1 and Cat1 (Nakase *et al.* 2013; Nakashima *et al.* 2014). Cat1 is the major cationic amino acid transporter in *S. pombe* , and loss of Cat1 results in canavanine resistance and a severe reduction of arginine uptake (Aspuria and Tamanoi 2008). Interestingly, despite sharing similar roles in arginine transport and canavanine toxicity, *S. pombe* Cat1 and *S. cerevisiae* Can1 are not orthologs (Figure 1D). Instead, they belong to two distinct subfamilies that have each undergone lineage-specific expansion (Figure 1D). Consistent with the function of Any1 in downregulating Cat1, deletion of *any1* renders *S. pombe* cells more sensitive to canavanine, whereas overexpression of *any1* confers canavanine resistance (Nakase *et al.* 2013; Nakashima *et al.* 2014). Because the *any1-R175C* ( *can1-1* ) mutation shares the same phenotypic consequence as the overexpression of *any1* , we propose that the *any1-R175C* mutation is likely a gain-of-function mutation.

To explore the possible reason behind the gain-of-function effect of the *any1-R175C* mutation, we examined how Any1 may interact with Cat1 by applying the protein complex structure prediction tool AlphaFold-Multimer (Evans *et al.* 2021). The prediction result shows that a short stretch of 10 amino acids (residues 43-52) in the N-terminal cytoplasmic tail of Cat1 interacts with the arrestin-N domain of Any1 (Figure 1E). This stretch is unstructured (pLDDT < 50) in the AlphaFold-predicted structure of Cat1 alone (Jumper *et al.* 2021; Varadi *et al.* 2022). Intriguingly, the R175 residue of Any1 is located at the predicted interaction interface. This prompted us to predict the structure of the Any1-R175C mutant protein in complex with Cat1. Remarkably, in the mutant complex, three more residues of Cat1 (residues 40-42) become structured and make contacts with Any1 (Figure 1E). The interface area increases from 578.6 Å ^2^ in the wild-type complex to 739.2 Å ^2^ in the mutant complex, and the free energy of dissociation (energy required to dissociate the two proteins) increases from 2.0 kcal/mol for the wild-type complex to 3.7 kcal/mol for the mutant complex, suggesting that the R175C mutation enables Any1 to interact more strongly with Cat1. These structure prediction results support a model that the *any1-R175C* ( *can1-1* ) mutation is likely a gain-of-function mutation that results in stronger downregulation of Cat1 (Figure 1F).

The proposed gain-of-function nature of the *any1-R175C* ( *can1-1* ) mutation is consistent with the fact that *can1-1* is a semi-dominant mutation in that *can1-1* / *can1 ^+^* heterozygous diploid cells are substantially more resistant to canavanine than *can1 ^+^* / *can1 ^+^* diploid cells and slightly less resistant than *can1-1* / *can1-1* diploid cells (Fantes and Creanor 1984). This is different from the situation in *S. cerevisiae* , where all canavanine-resistant mutations (loss-of-function *can1* mutations) are recessive (Whelan *et al.* 1979). The reversal of the canavanine resistance phenotype of the *can1-1* mutant by the expression of the *S. cerevisiae* *CAN1* gene (Ekwall and Ruusala 1991) can be explained by an inability of Any1-R175C to downregulate *S. cerevisiae* Can1, as the Any1-interacting region of *S. pombe* Cat1 predicted by AlphaFold-Multimer is not conserved in *S. cerevisiae* Can1.

The frequency of canavanine-resistant mutations has been used as a readout of mutation rate in *S. pombe* (Kaur *et al.* 1999; Fraser *et al.* 2003; Tanaka *et al.* 2004; Sabatinos *et al.* 2013). The clarification of the identity of the gene affected by the *can1-1* mutation will benefit the design and interpretation of canavanine resistance-based analyses of mutation rates and mutation spectra. Loss-of-function mutations in the *cat1* gene are likely to be much more common than gain-of-function mutations in the *any1* gene among spontaneous and mutagen-induced canavanine-resistant *S. pombe* mutations.

## Methods

**Whole genome sequencing and sequencing data analysis:** Tagmentation-based sequencing library preparation was performed as described previously (Tao *et al.* 2019). Paired-end 2 × 150 bp reads were generated on the Illumina NovaSeq 6000 platform. FASTQ files were pre-processed by cutadapt (version 3.3) with the options –gc-content=36 -q 30,30 –trim-n -m 50 –pair-filter=any (Martin 2011). Potential cross-contaminating reads that harbor 27-mers with frequencies lower than 20% of the peak frequency of 27-mers were identified and filtered out using KAT (Mapleson *et al.* 2017). The pre-processed and filtered reads were mapped to the *S. pombe* reference genome (https://www.pombase.org/data/releases/pombase-2021-03-01/) using BWA-MEM (version 0.7.17) (Li 2013). The Genome Analysis Toolkit (GATK, version 4.2.0.0) was used for converting SAM files to BAM files, removing duplicate reads, and variant calling (McKenna *et al.* 2010). The read mapping results were visualized using JBrowse 2 (Hershberg *et al.* 2021). Hard-filtering thresholds recommended by the GATK team were used to filter false variants. SnpEff (version 5.0) was used for annotating variants (Cingolani *et al.* 2012). The sequencing data have been deposited under the Project ID CNP0002483 at the sequence archive of China National GeneBank (CNGB) (https://db.cngb.org/search/project/CNP0002483).

**Transformation of *any1* PCR products:** Primers 5′-gcactacctcgttcaaccac-3′ and 5′-tgtttcccgcaacgttcttt-3′ were used to perform PCR amplification from genomic DNAs of a wild-type strain and a *can1-1* strain. The PCR products are 934-bp long and encompass most of the coding sequence of the *any1* gene. The mutated nucleotide in the *any1-R175C* PCR product amplified from the *can1-1* strain is 507 bp away from one end of the PCR product and 426 bp away from the other end. A wild-type strain, LD331, was transformed separately with equal amounts of the two PCR products. For each transformation, 0.2 OD600 units of transformed cells were plated on an EMM plate containing 100 μg/ml of canavanine and incubated at 30 °C for 7 days. Images of plates were taken by scanning using an Epson Perfection V800 Photo scanner.

**Phylogenetic analysis:** The sequences of G3DSA:1.20.1740.10 domain-containing proteins were retrieved using Ensembl BioMarts (Kinsella *et al.* 2011). Sequences were aligned using the online MAFFT server (https://mafft.cbrc.jp/alignment/server/) (Katoh *et al.* 2019). The E-INS-i iterative refinement algorithm of MAFFT was used. A maximum likelihood tree was calculated using IQ-TREE version 2.1.3 for Mac OS X (Minh *et al.* 2020). The tree was rooted by midpoint rooting and visualized using FigTree version 1.4.4 (http://tree.bio.ed.ac.uk/software/figtree/).

**Protein complex structure prediction:** The structures of Any1-Cat1 complexes were predicted using AlphaFold-Multimer with default parameters (Evans *et al.* 2021). Full-length protein sequences were used for prediction. The predicted structures were visualized using PyMOL (version 2.5.0). Among the 5 structures predicted using the 5 different trained models of AlphaFold-Multimer, we chose the one with the highest confidence score. The interface area and the free energy of dissociation of each complex composed of residues 17-338 of Any1 and residues 40-52 of Cat1 were calculated using the PDBePisa web server (https://www.ebi.ac.uk/pdbe/pisa/) (Krissinel and Henrick 2007).

## Reagents

**Table:** 

Strain	Genotype	Original strain name	Original source	NBRP ID
DY7144	*h* ^−^ * can1-1*	Kohli 10-389	Jürg Kohli	FY18665
DY8198	*h* ^+^ * ade7-50 his3-D1 can1-1 leu1-32*	KS3402	Ken Sawin lab (Anders *et al.* 2008)	–
DY8199	*h* ^−^ * ade7-50 his3-D1 can1-1 leu1-32*	KS3404	Ken Sawin lab (Anders *et al.* 2008)	–
DY49190	*h* ^−^ * leu1-32 ura4-D18*	PR109	Paul Russell lab	–
DY49191	*h* ^+^ * leu1-32*	KS1599	Paul Russell lab	–
LD331	*h* ^+^	–	Li-Lin Du	–
